# GRACE: Graph autoencoder based single-cell clustering through ensemble similarity learning

**DOI:** 10.1371/journal.pone.0284527

**Published:** 2023-04-14

**Authors:** Jun Seo Ha, Hyundoo Jeong

**Affiliations:** 1 Artificial Intelligence Graduate School, Gwangju Institute of Science and Technology, Gwangju, South Korea; 2 Department of Mechatronics Engineering, Incheon National University, Incheon, South Korea; Mohammed VI Polytechnic University, MOROCCO

## Abstract

Recent advances in single-cell sequencing techniques have enabled gene expression profiling of individual cells in tissue samples so that it can accelerate biomedical research to develop novel therapeutic methods and effective drugs for complex disease. The typical first step in the downstream analysis pipeline is classifying cell types through accurate single-cell clustering algorithms. Here, we describe a novel single-cell clustering algorithm, called GRACE (GRaph Autoencoder based single-cell Clustering through Ensemble similarity larning), that can yield highly consistent groups of cells. We construct the cell-to-cell similarity network through the ensemble similarity learning framework, and employ a low-dimensional vector representation for each cell through a graph autoencoder. Through performance assessments using real-world single-cell sequencing datasets, we show that the proposed method can yield accurate single-cell clustering results by achieving higher assessment metric scores.

## Introduction

Single-cell sequencing provides effective means to estimate gene expression profiles for individual cells so that it can help deciphering complex biological mechanisms underlying each cell [1–5]. Compared to the next-generation sequencing, where it can only capture the averaged gene expression profiles of cells in a tissue, the distinctive advantages of the single-cell sequencing can be more appealing methodology for biomedical researchers to expedite developing novel drugs and effective therapies for complex disease such as cancer and neurodegenerative disease.

Although single-cell sequencing techniques have attractive features for advanced biomedical research, there are critical drawbacks. Since it can capture the cell specific gene expression profile through the cell dissection and isolation process, it cannot provide the cell type labels for each cell, where it is crucial information in order to interpret a dynamic nature and heterogeneity of biological mechanisms across different cell types. Due to the inherent limitation of single-cell sequencing techniques, the typical first step of a downstream analysis in the analysis pipeline is predicting cell types for each cell in the sequencing result [6–10]. However, since the current single-cell sequencing can simultaneously profile the gene expression of the thousands of (or millions of) cells per experiment, it is challenging to manually annotate the cell types for numerous cells so that fully automated computational approaches would be the desirable and cost-effective way to deal with large-scale single-cell sequencing results. The general approach to annotate cell type labels includes two steps: i) prediction for the clusters of single-cells and ii) identification of cell types by using auxiliary information such as cell type specific marker genes [11–13]. Hence, the accuracy of the cell type identification and consequential downstream analysis highly depends on the quality of the single-cell clustering results, where it motivates for developing sophisticated single-cell clustering algorithms.

To enhance the accuracy of the cell type identification process, several single-cell clustering algorithms have been proposed based on different strategies and distinctions. Seurat [14] constructs a *K*-nearest neighbors network based on the similarity between 10 principal components for each cell. Then, it derives the clustering labels for each cell by optimizing a modularity through Louvain algorithm [15]. To the best of our knowledge, CIDR [16] is the first single-cell clustering algorithm that adopts the zero-inflated noise reduction module in the clustering algorithm. It first reduces the artificial zeros in a single-cell sequencing data and it estimates the dissimilarity among cells. Then, CIDR obtain the single-cell clustering through a hierarchical clustering. SC3 [17] measures similarities between cells through Euclidean distance, Pearson and Spearman correlation. Next, it transforms the similarity measurements into the normalized Laplacian and initial clustering through *k*-means clustering based on different number of eigen vectors. Finally, SC3 obtains the consensus matrix through cluster-based similarity partitioning algorithm [18] and derive the clustering labels through a hierarchical clustering. pcaReduce [19] first obtains the naive single-cell clustering through *K*-means clustering algorithm through principal components for each cell. Then, pcaReduce repeatedly merges a pair of clusters with the highest similarity until it attains the user-defined number of clusters. SinNLRR [20] estimates the cell-to-cell similarity through the low-rank representation of each cell. In order to obtain the low-rank representation for each cell, SinNLRR constructs the optimization problem based on the assumption that the gene expression of one cell can be derived through the mixture of gene expression of cells in the same cell type. Once it obtains the low-rank representation of each cell, SinNLRR derive the accurate single-cell clustering through the spectral clustering [21]. scGNN [22] constructs KNN (*K*-nearest neighbors) graph based on the Eculidean distance of gene expression profiles between cells in order to represent the cell-to-cell similarities. Then, scGNN refines the cell similarity graph by pruning less relevant neighboring nodes (i.e., cells) using the isolation forest algorithm [23]. Finally, it iteratively employs three multi-modal autoencoders to derive accurate single-cell clustering results. scDSSC [24] also utilizes an autoencoder in order to obtain the low-dimensional embeddings for each cell. To derive the proper loss function for the autoencoder, scDSSC adopts the self-expressiveness property, where the gene expression for a cell can be represented as a linear combination of the gene expressions of other cells [25]. Once scDSSC obtains the low-dimensional vectors for cells, the accurate single-cell clustering can be derived through a spectral clustering algorithm.

In this work, we propose a novel single-cell clustering algorithm, called GRACE (**GR**aph **A**utheocoder based single-cell **C**lustering through **E**nsemble similarity learning). The proposed method adopts an ensemble similarity learning framework in order to avoid solving the optimal feature selection problem and derive the accurate cell-to-cell similarity measurement. Moreover, the proposed method leverages the graph autoencoder to obtain effective low-dimensional vector representations for each cell, where it can be applied to various single-cell analysis algorithms. First, in order to reduce a computational complexity and improve the reliability of the cell-to-cell similarity estimation, we collect a set of potential feature genes based on the variance of gene expressions across cells. Next, we iteratively estimate the cell-to-cell similarities through the different subsets of potential feature genes in order to increase the diversity of the similarity measurements. Then, we construct the ensemble cell-to-cell similarity network by integrating multiple similarity estimates that are derived through different feature sets. We obtain the low-dimensional vector representations (i.e., node embedding) of each cell by applying the ensemble cell-to-cell similarity network into a graph autoencoder. Finally, based on the low-dimensional vector representations for each cell, where it can be derived through the graph autoencoder, we estimate the number of clusters in a single-cell sequencing and obtain an accurate single-cell clustering labels through the *k*-means clustering algorithm. Based on the comprehensive assessments using real-world single-cell sequencing datasets, we demonstrate that GRACE can yield an accurate and reliable clustering results.

## Materials and methods

### Motivation and overview

To obtain in-depth analysis results of a single-cell sequencing data and decipher complex biological mechanisms underlying gene expression patterns, an effective single-cell clustering is an essential first step [6–10]. Although an accurate cell-to-cell similarity measurement plays a pivotal role in developing effective single-cell clustering algorithms, there are several hurdles for precisely estimating cell-to-cell similarities. First, although the domain knowledge helps accurately estimating cell-to-cell similarities that can lead to a development of effective single-cell clustering algorithms, it generally needs priceless resources such as human labour and cost to generate the biological prior knowledge or domain knowledge may not available for some cases. That is, if we can exploit a prior information such as marker genes, where it is exclusively expressed only in a particular cell type, the optimal cell-to-cell similarity can be efficiently derived. However, in a practical point of view, since a prior knowledge is typically unknown and marker genes can be identified through biological experiments that require a huge amount of valuable resources such as cost and time, it is challenging to directly employ these marker genes to develop single-cell clustering algorithms. Next, although it can be a reasonable alternative to estimate cell-to-cell similarities based on the feature genes that can describe the unique properties of a particular cell type rather than employing marker genes, we need to define a tailored cost function to select an effective set of feature genes. It is still challenging to determine the set of the optimal feature genes because the mathematical soundness may not guarantee the biological soundness even if we can define the optimal cost function for identifying the set of optimal feature genes in terms of mathematical perspectives. Furthermore, when considering the scale of current single-cell sequencing protocols, it is still challenging to define the optimal (or effective) cost function that can account a biological variability across a larger number of cells. Finally, due to the technical limitation of sequencing protocols, single-cell sequencing includes a larger number of dropout events that can be modeled as a zero-inflated noise [26, 27] and these artificial zeros make it challenging to accurately estimate the cell-to-cell similarities.

**Fig 1:**
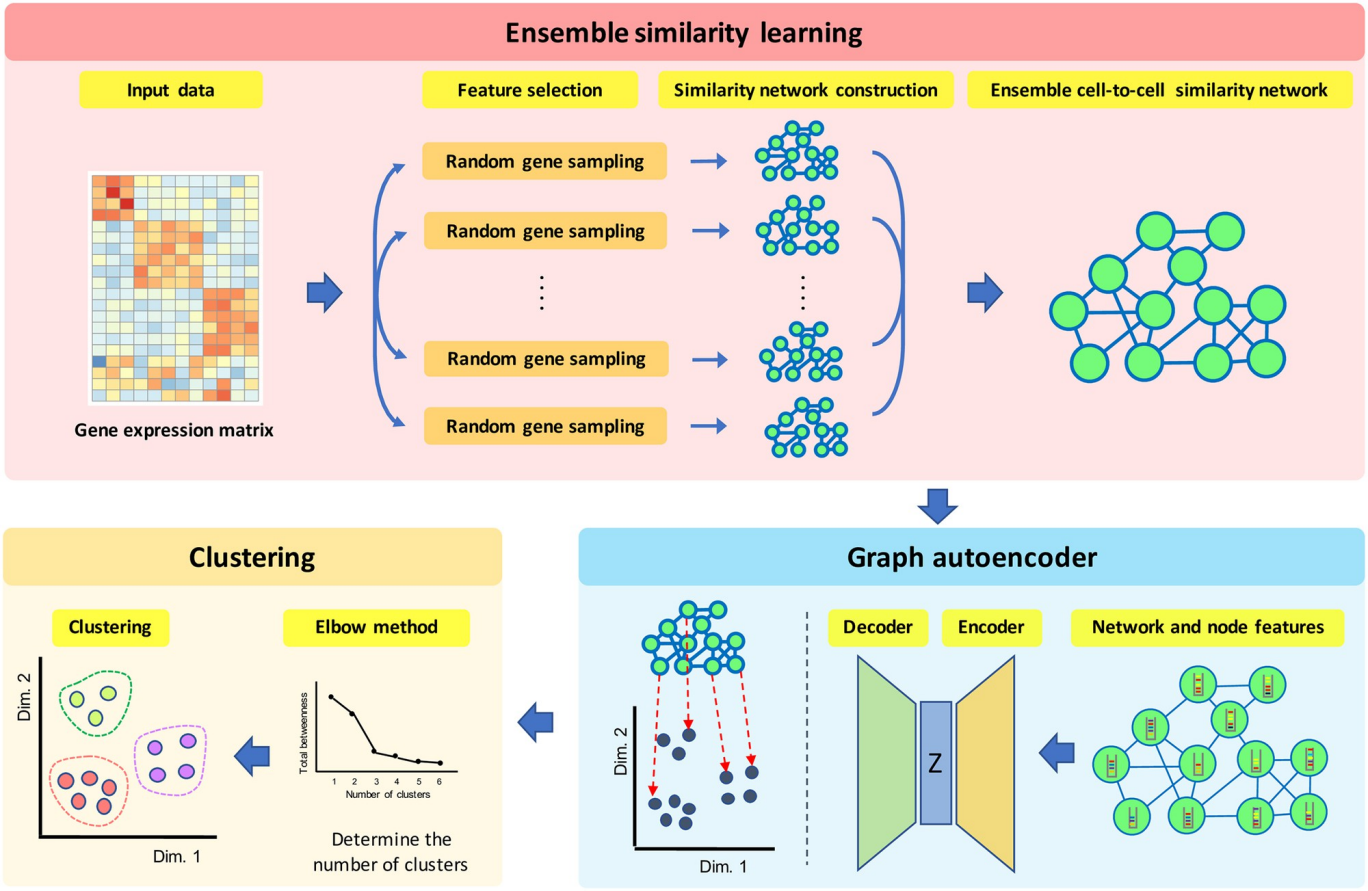
Graphical overview of the proposed single-cell clustering algorithm. GRACE includes three main steps to derive the accurate single cell clustering. First, the proposed method constructs the ensemble cell-to-cell similarity network, where it can effective represent the similarities between cells based on the multiple similarity measurements through different feature genes. Second, a graph autoencoder derives a low-dimensional vector representation for each node (i.e., cell). Finally, GRACE predicts the optimal number of clusters and yields the accurate single-cell clustering based on the low-dimensional vector representation for each node.

To overcome aforementioned technical challenges, we propose a novel single-cell clustering algorithm based on the ensemble similarity learning method and graph autoencoder. First, to obtain the reliable cell-to-cell similarity measurements without a biological prior knowledge such as cell type specific marker genes, we adopt an ensemble similarity learning framework, where it can obtain the reliable cell-to-cell similarities by incorporating a number of cell-to-cell similarity measurements based on different feature genes [28]. The key idea of the ensemble similarity learning approach is that, if two cells consistently achieve a high level of similarity even though it estimates the similarity based on different features (or similarity metrics), the two cells have a high probability to be classified into the same cell type. Based on the ensemble similarity learning framework, the cell type specific marker genes are not required and we can simultaneously avoid the optimal feature selection problem that requires defining the optimal cost function, but it can guarantee a decent performance by increasing the diversity of similarity measurements. Furthermore, although single-cell sequencing can include excessive zeros that can be modeled as zero-inflated noise, since the ensemble learning framework exploits multiple similarity measurements based on different feature sets for computing the cell-to-cell similarities, it can also mitigate the effect of zero-inflated noise. Then, after converting the estimated cell-to-cell similarities into a graphical model (i.e., ensemble cell-to-cell similarity network), we derive the effective low dimensional vector representations for each cell through a graph autoencoder. Finally, a node embedding vector can be directly utilized for determining the number of clusters and developing an accurate single-cell clustering algorithm. Based on the above solutions, the proposed method consists of three major steps: i) ensemble similarity learning for deriving a graphical model that can accurately represent cell-to-cell similarities, ii) deriving a low-dimensional vector representation (i.e., node embedding) of each cell through a graph autoencoder, and iii) clustering of single-cells based on the low-dimensional vector representation. Note that Fig 1 provides a graphical overview of the proposed single-cell clustering algorithm.

### Data normalization

Suppose that we have a single-cell sequencing data that can estimate gene expression profiles across cells. These gene expression values can be represented as a *M* by *N* dimensional matrix **X**, where *M* is the number of genes and *N* is the number of cells. In order to alleviate a technical bias, we perform a library size normalization [29, 30]. Although there are various normalization methods having distinctive advantages and we need to carefully exploit a sophisticated normalization method that is tailored to a particular sequencing platform, we employ a simple normalization method called the count per million (cpm), where it results the same library size for each sample (i.e., cells), because a selection of normalization methods is beyond the scope of this paper. After performing a library size normalization, since the distribution of gene expression in a single-cell sequencing data has a wide range and skewed numerical values, we also take a log-transformation in order to obtain a balanced range of expression values. Finally, we have the normalized gene expression matrix **X**_**n**_, where it is given by

$$\begin{array}{c} X_{n}={\text{log}}_{2}\left(1+X\right). \end{array}$$ (1)

### Estimation of a cell-to-cell similarity through ensemble similarity learning

We supposed that the accurate estimation of the cell-to-cell similarity (or correspondence) is the most important factor to yield reliable single-cell clustering results. To obtain the accurate estimation of a cell-to-cell similarity, we adopt the ensemble similarity learning strategy [28]. The fundamental assumption of the ensemble similarity learning approach is that, if two cells consistently achieve a high similarity score based on the diverse similarity measurements according to different feature sets, the two cells can have a high chance to be classified into the same cell type. To accommodate the ensemble similarity learning framework into the proposed single-cell clustering algorithm, we obtain multiple estimations of a cell-to-cell similarity through different similarity measurement methods based on different feature sets. Then, to obtain the accurate cell-to-cell similarity, we integrate multiple similarity measurements in a balanced manner, where it can also reduce a potential sampling bias.

Fist, we identify the feature gene candidates, where it can have a great potential to be a marker gene for a particular cell type. Note that marker genes are typically highly expressed in a specific cell type and rarely expressed in the rest of cell types. Additionally, our goal is not precisely identifying the marker genes and it would be acceptable if we can collect a set of genes that can have a discriminative power for separating different cell types. To collect the feature gene candidates, we compute the variance of each gene across whole cells and collect the top five percent genes having the largest variances, where it can be a set **F** of feature gene candidates. Next, we repeatedly estimate the cell-to-cell similarities based on the subset of feature gene candidates (i.e., **f** ⊂ **F**). That is, to determine the *l*-th similarity measurement, we obtain a subset **f**^*l*^ of feature genes based on the random sampling strategy. Note that, for each random sampling process to select the *l*-th subset **f**^*l*^, the seventy percent of genes are randomly extracted from the feature gene candidates **F**. Then, we compute the cell-to-cell similarity based on the correspondence between cells, where it can be determined by both clustering algorithms and a low-dimensional representation for each cell that can be derived through t-SNE [31]. If two cells are grouped together in a low dimensional space, we consider that the two cells could belong to the same cell type (i.e. they are similar to each other), otherwise the two cells could not be similar to each other. Note that, to enhance diversity of similarity measurements, we adopt different clustering algorithms such as *k*-means and hierarchical clustering algorithms because each algorithm can have a particular strength to capture different geometrical characteristics of a data distribution.

Based on the cell-to-cell correspondence estimation through *k*-means clustering algorithm over the low-dimensional space, the *l*-th similarity estimation can be represented a matrix **K**^*l*^, where it is given by

$$\begin{array}{c} K^{l}[i,j]=\{\begin{array}{cc} 1, & c_{j}\in N_{K}(c_{i}),\forall c_{i}\\ 0, & o.w. \end{array}, \end{array}$$ (2)

where *K*^*l*^[*i*, *j*] is an element in *i*-th row and *j*-th column of the matrix **K**^*l*^ and $N_{K}(c_{i})$ is a set of cells that are grouped together with the *i*-th cell *c*_*i*_ through *k*-means clustering algorithm. Note that *i*-th row and *j*-th column in the matrix **K**^*l*^ indicate the similarity between *i*-th cell *c*_*i*_ and *j*-th cell *c*_*j*_.

Similarly, a hierarchical clustering algorithm over the low-dimensional space can determine the *l*-th similarity estimation that can be represented as a matrix **H**^*l*^, where it is given by

$$\begin{array}{c} H^{l}[i,j]=\{\begin{array}{cc} 1, & c_{j}\in N_{H}(c_{i}),\forall c_{i}\\ 0, & o.w. \end{array}, \end{array}$$ (3)

where *H*^*l*^[*i*, *j*] is an element in *i*-th row and *j*-th column of the matrix **H**^*l*^ and $N_{H}(c_{i})$ is a set of cells that have the same clustering label to the *i*-th cell *c*_*i*_ through a hierarchical clustering. Additionally, *i*-th row and *j*-th column in the matrix **H**^*l*^ indicate the similarity between *i*-th cell *c*_*i*_ and *j*-th cell *c*_*j*_. Note that, we empirically set the number of clusters as 30 for each similarity measurement using the *k*-means and hierarchical clustering. Since the goal of clustering for measuring the cell-to-cell similarity is identifying highly consistent group of cells, even though cells in the same type would be divided into different subgroups, it would be much appropriate to derive a larger number of clusters to make homogeneous cell groups.

Finally, we integrate two matrices **K**^*l*^ and **H**^*l*^ and repeat the similarity estimation process for a certain number of times in order to derive the ensemble cell-to-cell similarity **A**, where it is given by

$$\begin{array}{c} A=\sum_{l=1}^{L}(K^{l}+H^{l}). \end{array}$$ (4)

Note that we empirically set the number of ensemble learning process as 20 in order to mitigate the effect of a sampling bias and enhance the accuracy of the similarity estimation by increasing the diversity of measurements.

### Single-cell clustering through a graph autoencoder

To obtain accurate single-clustering results, we leverage a graphical representation of cells because a graphical model can provide an intuitive way of describing complex relationships among a number of objects. Moreover, we can take advantage of numerous mathematical theories and well-developed algorithms that are tailored to analyze graphical models [32–34]. To bring the advantages of graphical models into the proposed method, suppose that we have a graph G=(V,E,W), where the *i*-th cell can be represented as a node $v_{i}\in V$, and the edge $e_{i,j}\in E$ indicates the binary correspondence between *i*-th and *j*-th cells, and their similarity can be represented as a weight function W:E→R that can represent the level of the similarity between cells. Based on the graphical model, the ensemble cell-to-cell similarity measurement **A** can be considered as an adjacency matrix of the graph G.

Next, we adopt a graph autoencoder (GAE) in order to obtain a low-dimensional vector representation for each cell because GAE can effectively take both a topological structure (i.e., similarity relationships among cells) of the graph and features for each node into account to learn a low-dimensional vector representation [35]. In this work, the architecture of GAE consists of an encoder and a decoder, where the encoder has a graph convolutional network (GCN) with two layers that can yield a embedding for each node [36]. The encoder can yield the low-dimensional embedding **Z** for each node, where it is given by

$$\begin{array}{c} Z=GCN\left(X,A\right)=\tilde{A}ReLU\left(\tilde{A}XW_{0}\right)W_{1}, \end{array}$$ (5)

where **W**_*i*_ indicates the weight matrix for the *i*-th layer, $\tilde{A}$ is a symmetrically normalized adjacency matrix (i.e., $\tilde{A}=D^{-1/2}AD^{-1/2}$) and *ReLU*(*x*) is a rectifier linear unit function that gives *max*(0, *x*). Note that we set the initial values in the weight matrix **W**_*i*_ based on the method in [37].

In the decoder block, GAE reconstructs the adjacency matrix $\hat{A}$ through $\hat{A}=\sigma \left(ZZ^{T}\right)$, where *σ*(⋅) is a logistic sigmoid function. To learn the low-dimensional embedding **Z** for each node, GAE minimizes the reconstruction error between $\hat{A}$ and **A** by opmizing the objective function. Note that we adopt the same objective function in [35]. Additionally, to describe the feature **X** for each node, we employ the first 10 principal components for each cell because we suppose that first 10 PCs can effectively capture the enough variance for each cell. Finally, we set the number of hidden nodes in the first and second GCN layers as 32 and 16, respectively.

Based on the low-dimensional vector represemtaion derived by GAE, we estimate the number of clusters through the elbow method and we obtain the single-cell clustering labels through *k*-means clustering algorithm because, based on our experimental results, these combination provides promising performances. Note that the algorithm 1 summarizes the pseudo-code of the proposed method.

**Algorithm 1:** GRACE

**Data:** Single-cell sequencing data **X**

**Result:** Clustering labels for each cell

**begin**

 Data normalization and log-transformation using Eq (1) Select a set **F** of potential feature genes **for** *l* = 1 *to L* **do**

  **f**^*l*^ ⊂ **F** /* Random feature (gene) sampling   */

  **x** = *t*-SNE(**f**^*l*^) Perform *k*-means and hierarchical clustering Construct the similarity matrix **A**_*l*_ using Eqs (2) and (3)

 **end**

 $A=\sum_{l=1}^{L}(A^{l})$ /* Ensemble similarity matrix   */

 **Z** = *GCN*(*A*, *X*) /* Obtain a node embedding   */

 *k* = *elbow*(**Z**) /* Determine the number of clusters   */

 *cl* = *kmeans*(**Z**) /* Perform *k*-means clustering    */

**end**

## Results

### Datasets

We evaluated the effectiveness of the proposed single-cell clustering algorithm against the state-of-the-art algorithms through fourteen single-cell sequencing datasets. First, we accessed NCBI GEO (Gene Expression Omnibus) and downloaded a raw count (or relative gene expression) matrix. Next, we removed all genes that are not expressed across whole cells because these genes would not be necessary but it can increase a computational complexity and memory consumption in an algorithmic point of view. Then, we employed processed real-world single-cell sequencing datasets to compare the performance of single-cell clustering algorithms. Usoskin et al. [38] sequenced mouse sensory neurons and it provides gene expression values of four different cell types: peptidergic nociceptors (PEP), non-peptidergic nociceptors (NP), neurofilament containing(NF), and tyrosine hydroxylase containing (TH). Kolodziejczyk et al. [39] provided a gene expression for pluripotent cells under different environmental conditions. Klein et al. provided a single-cell sequencing data for mouse embryo stem cells [4]. Zeisel et al. [40] obtained the gene expression data for cells in the mouse somatosensory cortex and hippocampal CA1 region. Zeisel dataset includes seven major cell types, where it can be categorized into 47 different subtypes. Among seven major cell types, since the population of three cell types is relatively smaller than other types, we only retained the following major cell types: interneurons, oligodendrocytes, pyramidal CA1 and pyramidal S1 neurons. Baron et al. [41] performed single-cell sequencing for cells in human and mouse pancreatic islets. For the same reason, we only employed six major populations such as alpha, beta, delta, ductal, gamma, and acinar. Furthermore, for the sequencing data obtained from the mouse pancreatic islets, acinar cells are also excluded as its population is relatively smaller than other cell types. Manno et al. [42] provided the gene expression profile for cells in the ventral striatum (mouse brain). We downloaded the preprocessed data from PanglaoDB [43]. Although the preprocessed data includes total 7,788 cells, we only retained the cells having a cell type label and removed the cells without a specific label. We obtained the PBMC 8K data from the 10X single-cell sequencing database. The PBMC 8K data includes overall 8,381 cells that are obtained from the peripheral blood mononuclear cells. Wang et al. [44] sequenced T cells from the peripheral blood of healthy individuals (Wang_H) and cells in the B cell-acute lymphoblastic leukemia (B-ALL) patients (Wang_P). Although these datasets include overall 12,699 and 16,143 cells, respectively, we assessed the performance of each algorithm by sampling about 8,000 cells because of the limited computational resources. Table 1 summarizes the basic statistics of each single-cell sequencing data.

**Table 1:** Summary of single-cell sequencing datasets.

Dataset	#Cells	#Genes	#Clusters	Accession	Tissue
Usoskin	622	19,534	4	GSE59739	Mouse sensory neurons
Kolod	704	10,684	3	E-MTAB-2600	Mouse embryo stem cells
Klein	2,717	24,047	4	GSE65525	Mouse embryo stem cells
Zeisel	3,005	19,968	7	GSE60361	Mouse brain
Baron_h1	1,622	15,452	6	GSE84133	Human pancreas
Baron_h2	1,562	15,810	6	GSE84133	Human pancreas
Baron_h3	3,333	16,386	6	GSE84133	Human pancreas
Baron_h4	1,225	15,285	6	GSE84133	Human pancreas
Baron_m1	687	13,757	5	GSE84133	Mouse pancreas
Baron_m2	932	14,105	5	GSE84133	Mouse pancreas
Manno	6,980	27,845	13	PRJNA438862	Mouse Ventral striatum
PBMC_8k	8,381	33,694	11	10x Genomics Database	Peripheral blood mononuclear cells
Wang_H	8,234	13,466	9	GSE172158	Human peripheral blood
Wang_P	8,071	14,267	1	GSE172158	Human peripheral blood

### Parameter settings for each algorithm

We compared the performance of the proposed algorithm against cutting-edge single-cell clustering algorithms: Seurat [14], CIDR [16], SC3 [17], scGNN [22], and scDSSC [24]. To obtain single-cell clustering results for each algorithm, we employed the R packages for CIDR, SC3 and Seurat and python scripts for scGNN and scDSSC through the default or recommended parameter settings. Moreover, although the true number of clusters for each dataset is given, we employed the estimated number of clusters because the true number of clusters is typically unknown in a practical point of view. Note that, since each algorithm has a tailored method to estimate the true number of clusters, each method can yield different number of clustering labels. In the proposed method, we empirically set the model parameters. That is, we compared the simulation results through real-world single-cell sequencing datasets based on the diverse parameter settings and determined the following model parameters because it generally showed promising performances. To learn a low-dimensional vector representation for each node through GAE, we set the maximum number of iterations as 200 and selected the ADAM optimizer with a learning rate of 0.01. We performed all simulations using a desktop computer with Intel i5 processor having 12 cores, 48 GB system memory, and NVIDIA GTX 1060 GPU, where it is running on Windows 10 operating systems.

### Assessment metrics

To assess the performance of the clustering algorithms, we leveraged the external information such as the true cell type labels for each dataset. Based on the true cell type labels, we computed four different performance metrics: i) adjusted rand index (ARI), ii) normalized mutual information (NMI), iii) Jaccard index (JCCI), and iv) purity socre. To calculate the performance metrics, suppose that there are the true cell type labels $C=(c_{1},c_{2},\ldots ..,c_{K})$ for each dataset and we have the predicted clustering labels $Y=(y_{1},y_{2},\ldots ..,y_{J})$, where it can be obtained through each clustering algorithm.

First, the adjusted rand index is given by

$$\begin{array}{c} ARI=\frac{\sum_{ij}\left(\begin{array}{c} n_{i,j}\\ 2 \end{array}\right)-\left[\sum_{i}\left(\begin{array}{c} a_{i}\\ 2 \end{array}\right)\sum_{j}\left(\begin{array}{c} b_{j}\\ 2 \end{array}\right)\right]/\left(\begin{array}{c} n\\ 2 \end{array}\right)}{\frac{1}{2}\left[\sum_{i}\left(\begin{array}{c} a_{i}\\ 2 \end{array}\right)+\sum_{j}\left(\begin{array}{c} b_{j}\\ 2 \end{array}\right)\right]-\left[\sum_{i}\left(\begin{array}{c} a_{i}\\ 2 \end{array}\right)\sum_{j}\left(\begin{array}{c} b_{j}\\ 2 \end{array}\right)\right]/\left(\begin{array}{c} n\\ 2 \end{array}\right)}, \end{array}$$ (6)

where *n*_*i*,*j*_ is the number of cells that are assigned to the *i*-th predicted label even though their true label (i.e., the cell type) is the *j*-th label, *a*_*i*_ = ∑_*i*_(*n*_*ij*_), and *b*_*j*_ = ∑_*j*_(*n*_*ij*_).

Next, the normalized mutual information is given by

$$\begin{array}{c} NMI\left(Y,C\right)=\frac{2\times I(Y;C)}{H(Y)+H(C)}, \end{array}$$ (7)

where I(Y,C) represents the mutual information between Y and C, and H(Y) and H(C) are the entropies of labels Y and C, respectively.

The Jaccard index (JCCI) is given by

$$\begin{array}{c} JCCI\left(Y,C\right)=\frac{TP}{TP+FP+FN}, \end{array}$$ (8)

where TP is the number of correctly clustered cells, and FP is the number of cells in the same cluster with different true cell type labels, and FN is the number of cells that are assigned to the different predicted clustering labels but they have the same true cell type labels.

The purity score is given by

$$\begin{array}{c} Purity\left(Y,C\right)=\frac{1}{N}\sum_{J}{\text{max}}_{j}\left|y_{i}\cap c_{j}\right|, \end{array}$$ (9)

where *N* is the number of cells, and *J* is the number of predicted clustering labels.

We also compared the computational time of each clustering algorithm for different single-cell sequencing datasets in order to assess the scalability and computational complexity.

### Improved single-cell clustering through effective feature representations

**Fig 2:**
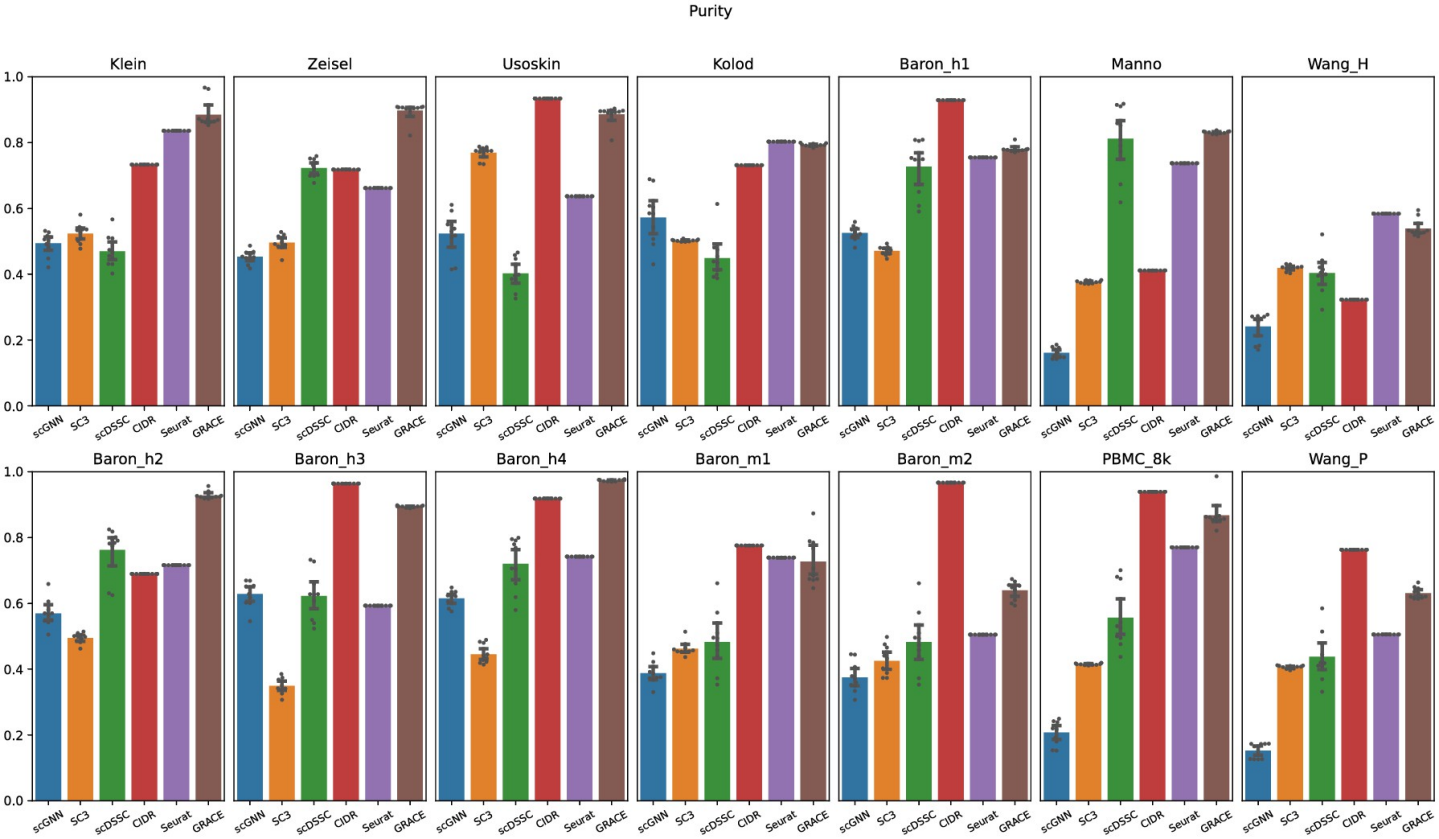
Purity scores for each clustering algorithm. Note that we performed 10 trials and visualize scattering points for each trial and the bar plot represents the averaged purity scores for all trials.

The aim of a single-cell clustering algorithm is identifying a homogeneous group of cells so that it can be employed to predict a cell type in a dataset without the help of biological validations, where it is a preliminary process in a single-cell analysis pipeline. To evaluate the consistency of the clustering results for each algorithm, we evaluated the purity scores for each algorithm. Among 14 single-cell sequencing datasets, GRACE achieved the highest purity scores for four datasets and attained the second-best purity scores for eight datasets (Fig 2). The average purity scores for scGNN, SC3, scDSSC, CIDR, Seurat, and GRACE were 0.422, 0.511, 0.575, 0.771, 0.685, and 0.805, respectively. Overall, although CIDR could be the strongest competitor for GRACE as it showed the higher or comparable purity scores, GRACE typically achieved the highest mean purity score over 14 datasets. In fact, CIDR includes the zero-inflated noise reduction process before deriving single-cell clustering results so that it can achieve the higher purity scores than the other algorithms if the single-cell sequencing includes larger number of artificial zeros. Based on our experimental results, we confirmed that GRACE can achieve the higher purity score even though it does not have a noise reduction step. This means that GRACE would have a possibility to further enhance the purity scores if it adopts the similar noise reduction module. Additionally, when comparing GRACE with other neural network based algorithms such as scGNN and scDSSC, the proposed method showed substantially higher purity scores.

**Fig 3:**
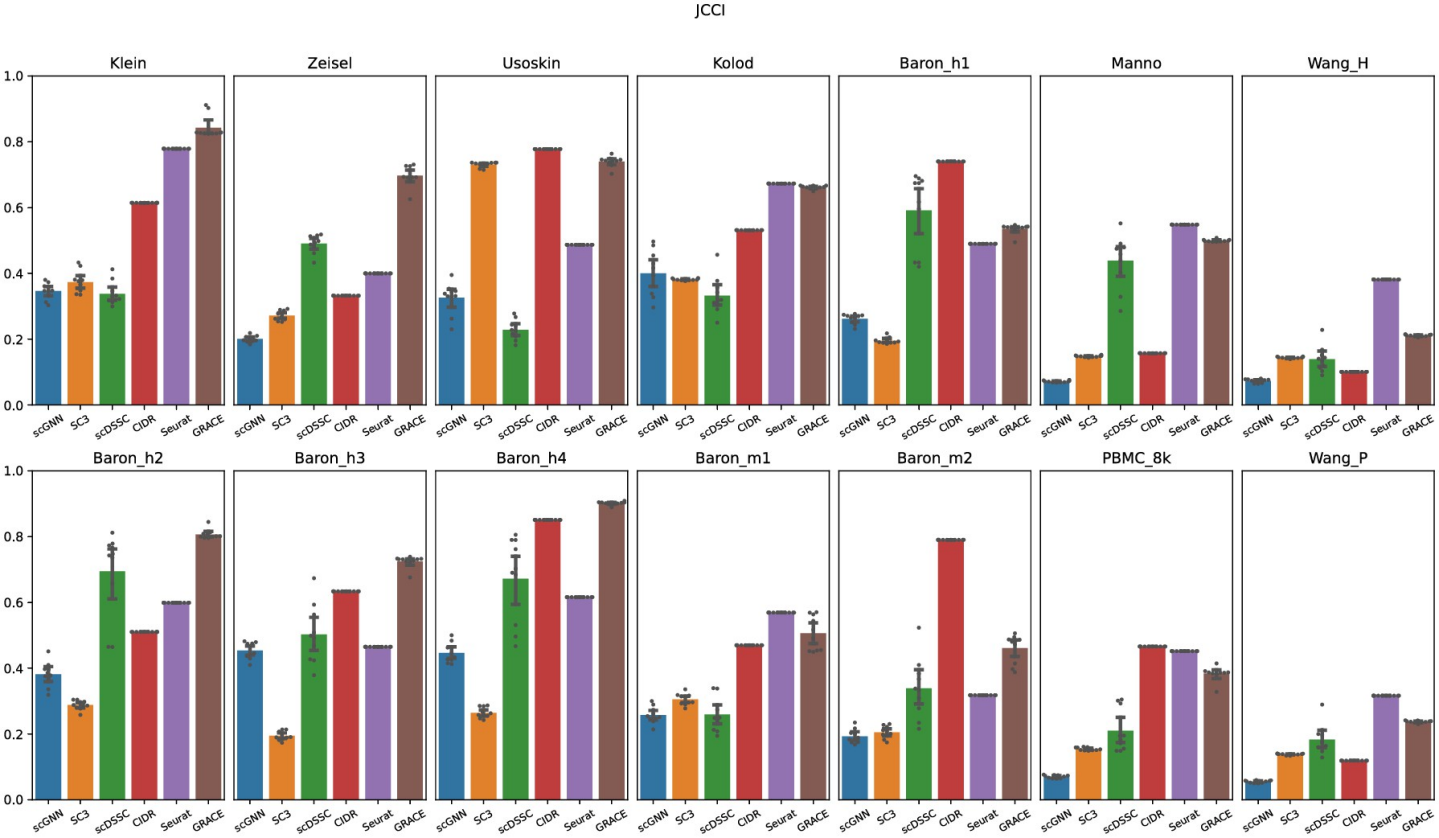
Jaccard index scores for each single-cell clustering algorithm. Note that scattering points represent the Jaccard index scores for 10 trials, and the bar plot represents the averaged Jaccard index score for all trials.

Next, we compared the Jaccard index for each clustering algorithm because, although the purity score can effectively assess the consistency of the clustering results, it has a risk to yield biased evaluations because the higher purity score can be easily achieved if the predicted clusters have a larger number of members. Given 14 experiments, we confirmed that GRACE achieved the best mean JCCI scores. Note that the averaged JCCI score for scGNN, SC3, scDSSC, CIDR, Seurat, and GRACE were 0.253, 0.271, 0.387, 0.507, 0.507, and 0.586, respectively (Fig 3). Especially, GRACE showed remarkably higher JCCI score for Klein, Zeisel, Baron_h2, Baron_h3, and Baron_h4 datasets. For instance, compared to the next-best algorithm for aforementioned five datasets, GRACE achieved about 15, 80, 40, 11, and 6 percent improvements. Although we confirmed that CIDR was the next best algorithm for the purity score, CIDR and Seurat achieved the same mean JCCI scores. Based on our experiments, CIDR tends to underestimate the number of clusters so that the number of clusters reported by CIDR is typically smaller than the true number of clusters and it naturally yields clustering results with a larger number of cells in each cluster. Hence, the purity score of CIDR may have a chance to be biased or overrated due to the size of the clustering results. Interestingly, Seurat showed the higher JCCI scores for the large-scale datasets including more than 6,000 cells. Since these four datasets are obtained 10X Genomics platform, we carefully deduced that Seurat could have a strength on the sequencing datasets from 10X Genomics platform. Additionally, although deep neural network based approaches show a favorable performance as the scale of dataset increases, scGNN and scDSSD showed relatively low JCCI scores even though they also adopt autoencoder. Since the single-cell sequencing datasets include the minor cell types, where the number of samples for minor types is typically smaller than the major cells, the sample imbalance could cause the degraded JCCI scores for two algorithms. We will discuss the sample imbalance in more detail (Discussion section).

**Fig 4:**
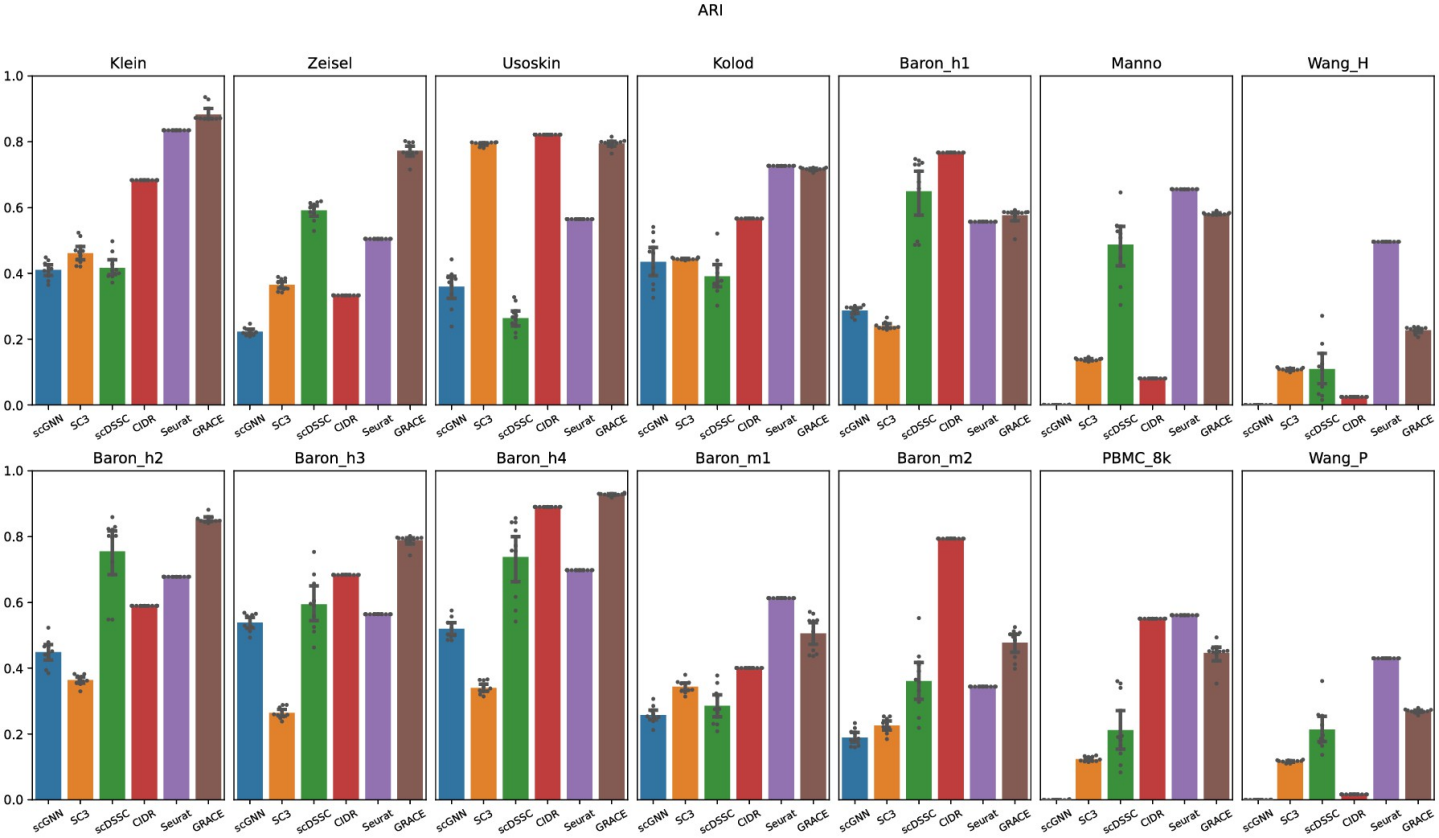
Adjusted rand index for each algorithm. Note that scattering points represent the ARI scores for 10 trials, and the bar plot represents the averaged ARI score for all trials.

**Fig 5:**
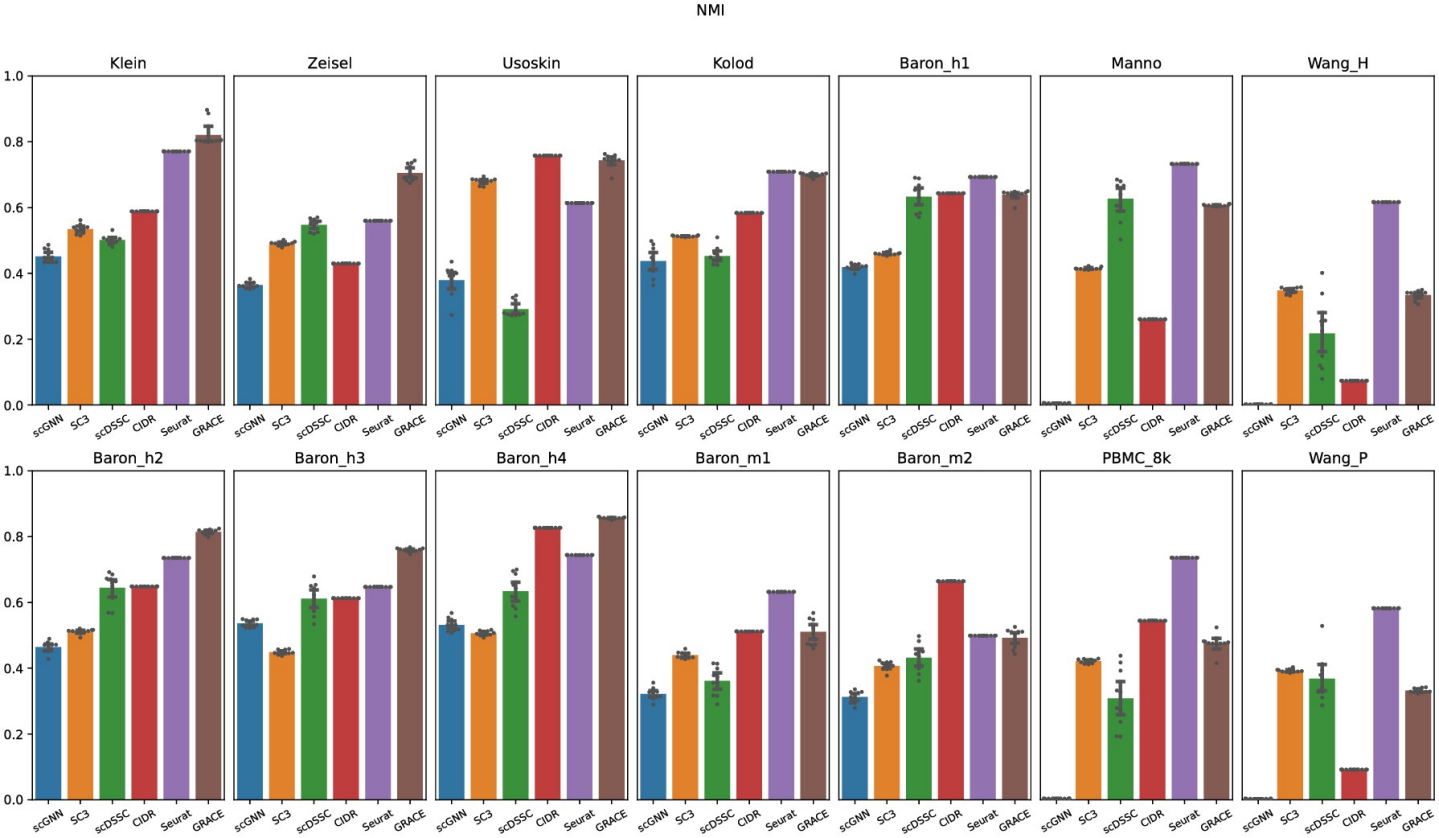
Normalized mutual information for each clustering algorithm. Note that scattering points represent the NMI scores for 10 trials, and the bar plot represents the averaged NMI score for all trials.

We also evaluated the quality of clustering results through the adjusted rand index to fairly show the effectiveness of the proposed algorithm. Although the Jaccard index score can provide reliable assessments for clustering algorithms because it takes a size factor of clustering results into account, Jaccard index score does not consider the true negatives. Note that, given two cells having a different true cell type labels in C, the true negatives count the case that the two cells are not assigned to the same predicted clustering label in Y. The averaged ARI for scGNN, SC3, scDSSC, CIDR, Seurat, and GRACE were 0.262, 0.309, 0.434, 0.514, 0.588, and 0.630, respectively (Fig 4). As we will show later, since SC3 overestimates the number of clusters, it achieved the least ARI scores across all datasets and this result apparently supports the importance of accurately estimating the number of clusters to yield reliable single-cell clustering results. Especially, only except the Usoskin and PBMC 8K datasets, GRACE achieved the highest or next-best ARI scores. Surprisingly, scGNN achieved very low ARI scores for the large-scale datasets. In these datasets, the number of major cells is much larger than the minor cells and these sample imbalance can cause a negative effect for constructing the cell-to-cell similarity graph by identifying KNN (K-nearest neighboring) cells, and inaccurate similarity graph can intervene the learning process of the graph autoencoder, where it could be the main reason resulting low ARI scores. Although GRACE adopts the graph autoencoder as well, it showed comparable ARI scores to Seurat because the ensemble similarity learning can accurately capture the cell-to-cell similarity correspondences and it can help the graph autoencoder to effectively capture the feature of each cell. Finally, we also compared the NMI for each clustering algorithm and we confirmed that it also showed the similar trends to other performance metrics (Fig 5). Overall, based on diverse performance metrics, we confirmed that GRACE outperforms the other competing algorithms, where it means that the proposed algorithm can yield more accurate single-cell clustering results compared to the state-of-the-art algorithms.

### Accurate prediction on the number of clusters

We compared the number of predicted clusters for each algorithm. In a practical point of view, the exact number of clusters for single-cell sequencing experiments is generally unknown and it is important to determine correct number of clusters for single-cell sequencing results in order to accurately annotate the cell types, where it is the major application of single-cell clustering algorithms. That is, if the predicted number of clusters is larger than the true number of clusters, cells in the same type can be divided into multiple subgroups. Additionally, if the predicted number of clusters is much smaller than the true number, cells in different types can be classified into the same partition. Although the accuracy of the clustering results is vulnerable to the predicted number of clusters, the importance of determining the number of clusters is easily overlooked when developing clustering algorithms.

**Fig 6:**
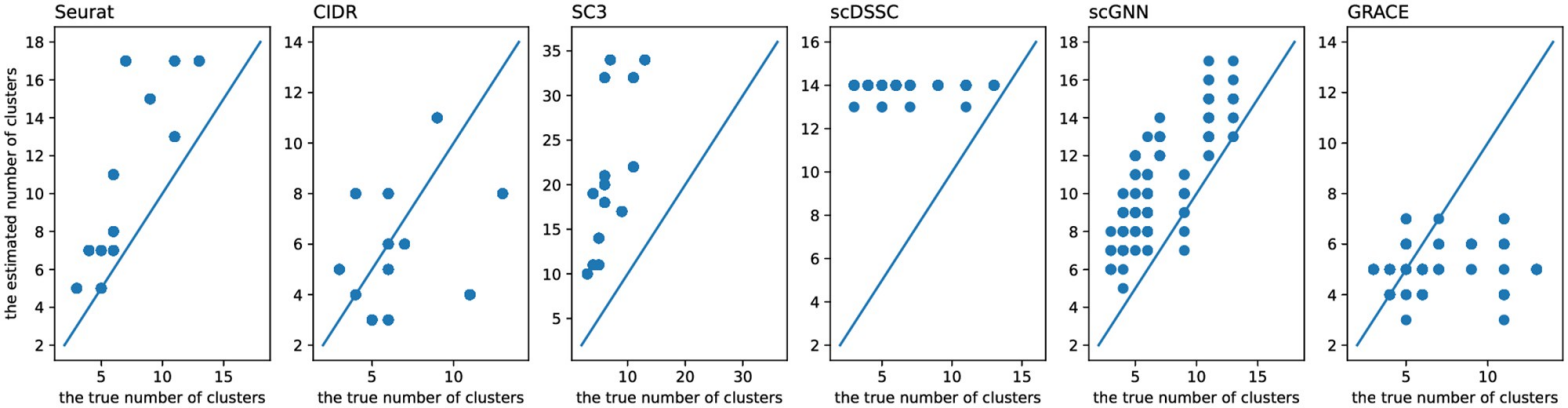
Comparison of the true and predicted number of clusters for each algorithm.

We compared the true and predicted number of clusters for 10 datasets (Fig 6). Note that, since each algorithm has a tailored method to estimate the number of clusters in the dataset, we employed a default setting in each algorithm to determine the predicted number of clusters. Additionally, since GRACE and scGNN have a stochastic nature when it estimates the cell-to-cell similarity through a random feature sampling, the number of predicted clusters could be different for each experiment and we showed all predicted results corresponding to the test cases in a single figure. Note that, to assess the performance of GRACE we measured the predicted number of clusters for 10 times. As we can see, SC3 typically overestimates the number of clusters so that it could have a risk to divide a consistent group of cells into multiple subgroups. It can affect the accuracy of the clustering results and it can be a possible answer for the low purity, JCCI, and ARI scores achieved by SC3. Similarly, scGNN also overestimates the number of clusters and it showed the larger standard deviations for the estimation results. For instance, the estimated number of clusters for the Wang_P datasets ranges 12 to 17 even though the true number of clusters is 11. For the same single-cell sequencing data, if the estimated number of clusters is spread across wide ranges, it is challenging to select the proper number of clusters. Seurat tends to identify more number of clusters compared to the true number of clusters for all test cases. Additionally, the estimation error of Seurat would increase as the true number of clusters increases, where it can cause more severe effects on the clustering results for single-cell sequencing having a larger number of subtypes. However, except large-scale datasets, GRACE showed the relatively smaller and balanced estimation error for most test cases. That is, except the single-cell sequencing datasets having more than 6,000 cells, the absolute differences between the true and predicted number of clusters were less than or equal to 2. Please note that, for the large-scale datasets that are sequenced from the peripheral blood mononuclear cells, they have multiple CD4 and CD8 subtypes that can be considered as a T-cell, and GRACE may not correctly separate these cell families so that it can cause relatively larger estimation error. Although GRACE showed relatively larger estimation error for the large-scale single-cell sequencing datasets, the average estimation error of GRACE is smaller than 3, where it is still smaller than the benchmarking algorithms. Note that, to quantitatively determine the estimation error, we computed the average estimation error through $\frac{1}{N}\sum_{n=1}^{N}\sum_{i=1}^{L}|K_{n}-x_{n,i}|$, where *N* is the number of datasets, *L* is the number of trials, *K*_*n*_ is the true number of clusters for *n*-th dataset, and *x*_*n*,*i*_ is the estimated number of clusters for the *n*-th datasest and *i*-th trial. The accurate prediction for the number of clusters can be a strong evidence for the effectiveness of both the ensemble similarity learning and low-dimensional representation through GAE that are core processes in the proposed algorithm.

### Separability of the clustering labels in a low-dimensional space

In order to intuitively analyze a large-scale single-cell sequencing data, visualizing cells in a two dimensional space is an effective and powerful approach [31, 45]. However, although there is the optimal low-dimensional representation method that can perfectly and clearly separate different cell types in a low-dimensional space, if there is no auxiliary information such as true cell types or if we have incorrect clustering labels, visualization of single-cells in a low-dimensional space does not provide meaningful insights for in-depth analysis.

To quantitatively evaluate the separability of cells in a two-dimensional space based on the predicted clustering labels, we assumed that, if the predicted clustering labels are correct or highly accurate, different cell types in a low-dimensional space can be clearly separated with an enough distance among different groups. Note that the goal of this assessment is comparing the performance of clustering algorithms without external information such as the true cell type labels. To compare the distance and separability between each group in a low dimensional space, we first derived the low-dimensional vector representation for each cell using UMAP [45], where it can generally provide a clear separation between different cell types. Next, we trained a linear SVM (support vector machine) based on the low-dimensional representation of randomly selected 80 percent cells and their predicted clustering labels. Note that, to train the linear SVM, we employed the same two vector dimensional representation (i.e., coordinates in a two dimensional space) for each cell but different clustering labels that are derived through different clustering algorithms. Then, based on the trained SVM, we classified the clustering labels for the rest of cells and determined the classification accuracy by comparing the classification results and the true cell type labels. In order to reduce the variance of the experiments, we perform the same experiments for 10 times and reported the averaged classification accuracy.

Based on 10 trials, we compared the averaged classification accuracy of cell types through a linear support vector machine (Table 2). Seurat achieved the highest accuracy for Kolod. data, and CIDR showed the best performance for Usoskin, Braon_h1, Braon_m1, Braon_m2, and Wang_P datasets. GRACE recorded the highest accuracy for eight datasets and attained the best runner-up for six datasets. On average, GRACE achieved the highest classification accuracy, where it is a clear evidence that GRACE can lead to an improved separability of cells in a low-dimensional space. Since the clear separability of cells in a low-dimensional space can help understanding large-scale single-cell sequencing datasets in an intuitive way, GRACE can provide pivotal the stepping-stones and insights for comprehensive analysis and understanding of single-cell sequencing results.

**Table 2:** Averaged classification accuracy of cells in a two dimensional space based on the predicted clustering labels. The two dimensional coordinates are derived through UMAP and cells are classified through a linear support vector machine. Note that linear SVM is trained through 10 different test cases and the averaged classification accuracy is compared. The highest value in each column is marked as a bold face character and the best runner up is highlighted as an underline format.

Methods	Klein	Zeisel	Usoskin	Kolod	Baron_h1	Baron_h2	Baron_h3	Baron_h4	Baron_m1	Baron_m2	Manno	PBMC 8K	Wang_H	Wang_P	Mean accuracy
scGNN	30.24	38.17	42.72	66.03	27.66	46.45	54.12	61.63	24.64	21.66	15.27	20.51	24.09	14.43	34.83
SC3	50.11	57.92	69.84	79.72	28.40	49.27	34.81	49.27	43.55	38.50	33.91	40.25	39.60	40.20	46.81
scDSSC	41.51	68.67	35.52	64.89	48.25	74.63	57.32	69.63	32.68	36.20	78.30	47.51	47.02	38.74	52.92
CIDR	47.79	71.38	**92.00**	97.16	**62.46**	64.86	72.41	87.35	**55.80**	**72.73**	39.83	72.09	47.54	**75.23**	68.47
Seurat	54.04	70.72	66.40	**98.58**	40.00	71.25	58.17	75.51	50.00	49.20	74.79	49.61	44.69	35.17	59.87
GRACE	**55.77**	**90.40**	87.68	97.30	48.37	**91.02**	**80.81**	**96.53**	55.65	61.50	**84.52**	**76.84**	**63.77**	61.37	**75.11**

**Fig 7:**
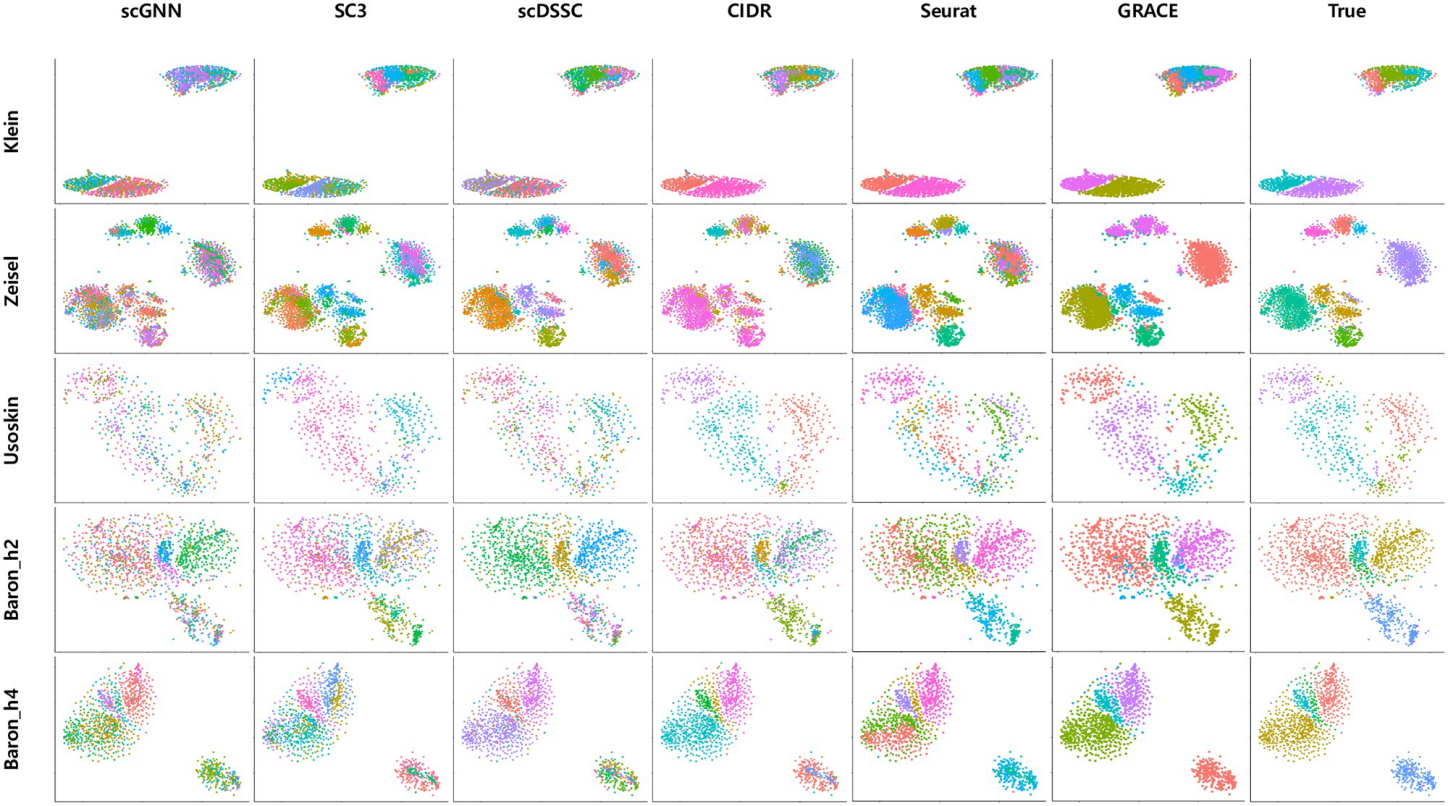
Low dimensional visualization of clustering results. Low-dimensional coordinates are derived through t-SNE and predicted clusters are annotated through different colors.

In order to intuitively verify the separability of clustering results for each algorithm, we plot the low dimensional visualizations for the clustering results through t-SNE (Fig 7). First of all, we obtained two dimensional coordinates for each dataset, and highlighted the predicted clustering results through different colors. Then, we compared the color annotations for each algorithm to the visualization results with the true cell type labels. For the Klein data, although GRACE and Seurat showed the good agreement to the ground truth, CIDR showed noticeable error for the cells that are located at the upper-right part. Other algorithms such as scGNN and scDSSC resulted prominent mismatches for most cell typtes. For the Zeisel data, except the visualization result of GRACE, two major cell types were divided into multiple clusters so that their annotation seems to be mixed by multiple color highlights. Note that, for the color annotation of the Zeisel data with the true cell type labels, two major cell groups are highlighted as purple and green colors. GRACE showed relatively good agreements to the ground truth and other visualization results also showed the similar trends.

### Computation time

**Fig 8:**
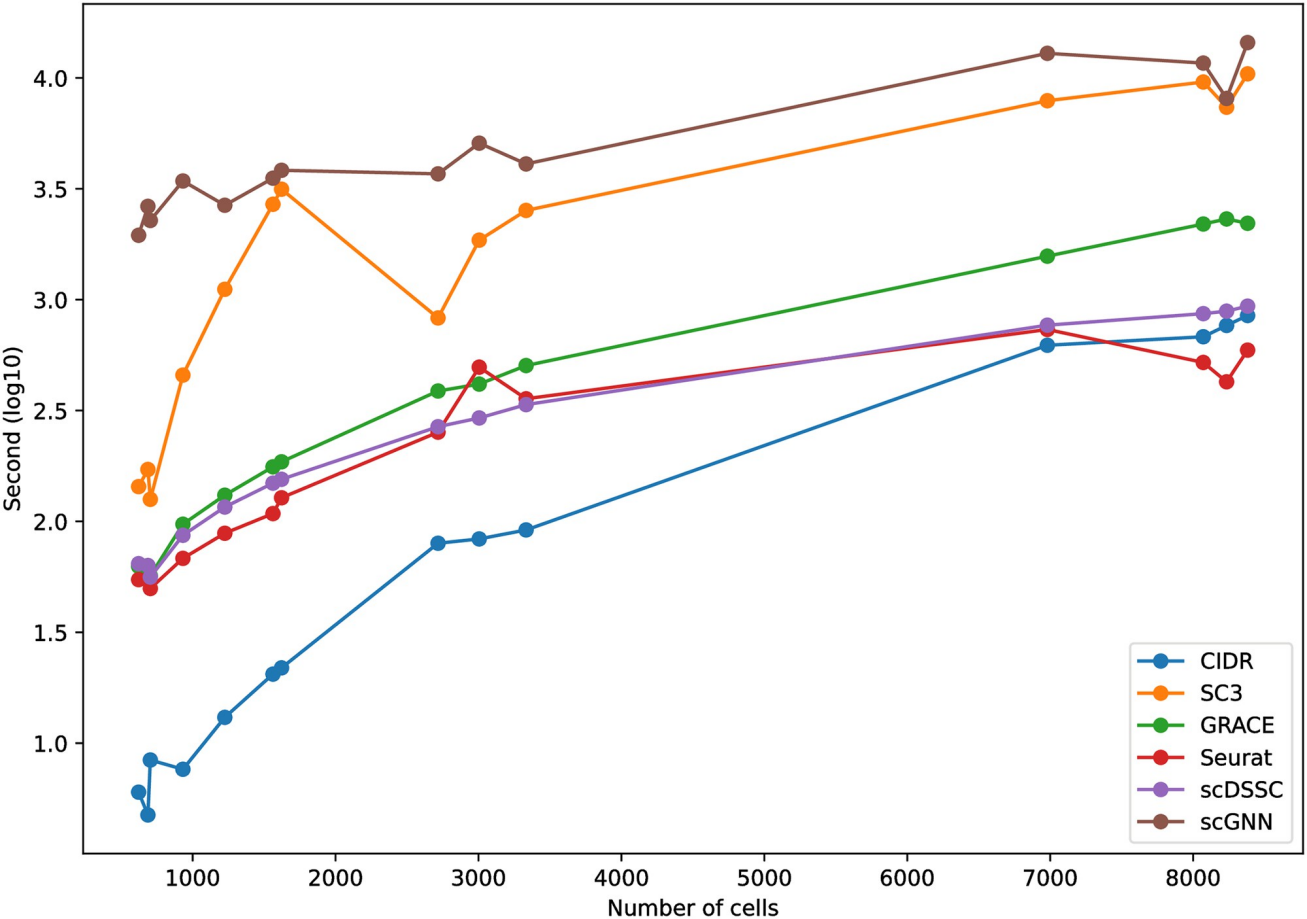
Computation time of each algorithm. All experiments were performed on Intel i5 processor with 12 cores, 48GB system memory, and NVIDIA GTX 1060 GPU. Note That the base clock frequency of the CPU is 4.10 GHz.

We compared the computation time for each clustering algorithm according to the number of cells in order to compare the scalability of algorithms (Fig 8). As we can see, scGNN requires the longest computation time for most cases because it integrates three multi-modal autoencoders in an iterative manner, where it naturally requires a huge amount of tensor computations. SC3 also needs relatively longer computation time for most cases because it requires a consensus clustering based on different similarity measurements. Although CIDR requires the least computation time for datasets having a relatively small scale, its computation time sharply increases as the scale of the datasets increases. That is, the scalability of CIDR would not be as good as other algorithms. Seurat showed the least computation time and superior scalability for most cases. Although GRACE requires slightly longer computation time compared to Seurat, it achieved an acceptable computation time and scalability even though it adopts ensemble similarity learning framework. Although scDSSC also adopts the autoencoder-based framework, its scalability would be slightly better than GRACE. Since GRACE constructs cell-to-cell similarity through ensemble similarity learning approach, it could be a bottleneck of the proposed method. However, GRACE still has a room for accelerating the computation time through parallel processing and powerful GPUs because the current version only employ a single core.

## Discussions and conclusion

We propose an effective single-cell clustering algorithm by leveraging the ensemble similarity learning framework and a graph autoencoder. First, in order to avoid the optimal feature gene selection problem, we collect a set of genes that can have a high probability to be a marker gene for each cell type based on a variance of the gene expressions across cells. Second, we determine multiple cell-to-cell similarity measurements based on the different subsets of the potential marker genes that can be obtained by a random gene sampling process. Next, we derive the accurate cell-to-cell similarity estimation by incorporating multiple similarity measurements in order to reduce a smapling bias. Based on the integrated similarity measurement, where it can be considered the graphical representation of cell-to-cell similarity, we obtain the low-dimensional vector representation for each cell through a graph autoencoder. Based on the low dimensional vector representation for each cell, we determine the number of clusters by using the elbow method and obtain the final single-cell clustering labels through the *k*-means clustering algorithm. Based on the real-world single-cell sequencing datasets, we confirm the effectiveness of the proposed single-cell clustering algorithm over the state-of-the-art algorithms.

The proposed single-cell clustering algorithm has several appealing advantages. First of all, it can avoid the optimal feature gene selection problem that is the essential and pivotal process to yield an accurate single-cell clustering. Second, thanks to the effective learning process in the graph autoencoder, the low-dimensional representation of each cell can be employed in the other single-cell analysis. Third, GRACE has a compatibility with other single-cell analysis algorithms because it does not require preprocessing steps to yield tailored data format. That is, since the proposed algorithm only requires a gene expression matrix, where it is typically required information for single-cell analysis pipeline, and it does not change the dimension of the input data, GRACE can be easily employed as a part of single-cell analysis pipeline. Furthermore, GRACE is a fully automated python script, where it does not require any biological domain knowledge such as cell type specific marker genes or the number of cell types. Since the prior knowledge is typically unknown before analyzing a single-cell sequencing data, the proposed algorithm is suitable for the first analysis step to derive a domain knowledge such as the number of cells in the dataset without biological experiments that require valuable resources such as cost and time.

Although the proposed method can yield accurate single-cell clustering results, there are unavoidable limitations. First, since the proposed method can only yield single-cell clusters that have a high probability to be the same cell type, it still requires additional biological cross validation such as verifying marker gene expression in order to clearly determine a specific cell type. Additionally, although it shows a moderate scalability and computational time, it still has a room to accelerate the computation speed through effective software implementation. More importantly, although the zero-inflated noise induced by dropout events has negative effects on the single-cell analysis results, the proposed method does not deal with the zero-inflated noise. To bridge these gaps, we would leverage a CPU parallel computing framework and a powerful GPU acceleration to reduce computational time and increase scalability of the method. To reduce the negative effect of zero-inflated noise on the analysis results, we would integrate a zero-inflated noise reduction module in the proposed algorithm. For instance, since an autoencoder is an effective method to reduce outliers such as the salt-pepper noise (i.e., extreme values) in images, we expect that the effective modification of the graph autoencoder can be a great candidate to remove such extreme values including artificial zeros.

In the future, we will consider the following research directions in order to develop an user-friendly single-cell analysis preprocessing pipeline. First of all, we will develop sophisticated single-cell data processing methods in order to increase the performance of the proposed method. That is, although GRACE adopts a graph autoencoder, where it typically shows improved performance as the scale of the dataset increases, we confirmed that the performance of autoencoder based algorithms such as GRACE and scGNN would not being surprisingly increased. Generally, a graph autoencoder effectively learns the hidden patterns underlying complex datasets when we have enough number of samples for each class. However, single-cell sequencing datasets includes minor cell types and the number of samples for the minor cells is typically much smaller than the major cells, and these sample imbalances can play as a hurdle to simultaneously take advantages of a graph autoencoder and large-scale datasets. To overcome the sample imbalance, we will examine the data augmentation methods that can generate artificial training datasets by taking the crucial features of single-cell sequencing data into account [46, 47]. Next, effective graph learning methods should be further investigated in order to increase the performance of graph-based clustering algorithms. Although both GRACE and scGNN adopt the graph autoencoder for deriving single cell clusters, they yield different performance metrics. One key difference between GRACE and scGNN is the method to construct the cell-to-cell similarity graph. That is, scGNN constructs KNN (K-Nearest neighbor) graph based on the Euclidean distance of the gene expression profile for each cell. Then, it refines the KNN graph by removing less-relevant neighboring cells through the isolation forest algorithm. However, GRACE constructs the cell-to-cell similarity graph through the ensemble similarity learning, where it can increase the diversity of similarity measurements. Based on the comparison results, we carefully conclude that the graphical representation is one of the pivotal step to develop a reliable and accurate graph based analysis algorithms. Recently, automated graph learning algorithms have been proposed and they show the promising results on diverse applications [48, 49]. Graph-based single-cell clustering algorithms can have increased performance headroom if we can integrate accurate graph learning algorithms. Moreover, since cell types can be classified into multiple categories, integrating multilayer graph clustering would be a reasonable alternative for the classical clustering algorithms such as *K*-means or spectral clustering algorithms [50–53]. In order to enhance the usability, it should be necessary endeavor for developing an effective graph clustering algorithm, where it can effectively take multiple subtypes of cells into consideration. Finally, to maximize the compatibility of the proposed algorithm, we would develop a comprehensive single-cell analysis pipeline based on an user-friendly cloud platform, where it can be employed diverse research groups without software experts or enough computing resources.
